# The type-I interferon response potentiates seeded tau aggregation and exacerbates tau pathology

**DOI:** 10.1002/alz.13493

**Published:** 2023-10-17

**Authors:** Sophie A. I. Sanford, Lauren V. C. Miller, Marina Vaysburd, Sophie Keeling, Benjamin J. Tuck, Jessica Clark, Michal Neumann, Victoria Syanda, Leo C. James, William A. McEwan

**Affiliations:** 1 UK Dementia Research Institute at the University of Cambridge, Cambridge, UK; 2 Department of Clinical Neurosciences, University of Cambridge, Cambridge, UK; 3 Medical Research Council Laboratory of Molecular Biology, Francis Crick Avenue, Cambridge, UK

**Keywords:** innate immunity, interferon, neuroinflammation, tau pathology, tauopathy

## Abstract

**Introduction:**

Signatures of a type-I interferon (IFN-I) response are observed in the *post mortem* brain in Alzheimer’s disease (AD) and other tauopathies. However, the effect of the IFN-I response on pathological tau accumulation remains unclear.

**Methods:**

We examined the effects of IFN-I signaling in primary neural culture models of seeded tau aggregation and P301S-tau transgenic mouse models in the context of genetic deletion of the IFN-I receptor (IFNAR).

**Results:**

Polyinosinic:polycytidylic acid (PolyI:C), a synthetic analog of viral nucleic acids, evoked a potent cytokine response that enhanced seeded aggregation of tau in an IFN-I-dependent manner. IFN-I-induced vulnerability could be pharmacologically prevented and was intrinsic to neurons. Aged P301S-tau mice lacking *Ifnar1* had significantly reduced tau pathology compared to mice with intact IFN signaling.

**Discussion:**

We identify a critical role for IFN-I in potentiating tau aggregation. IFN-I is therefore identified as a potential therapeutic target in AD and other tauopathies.

## 1 Background

Type I interferons (IFN-I) are a group of soluble cytokines that are produced following the detection of molecular patterns common to viruses. In humans, the group consists of IFN-*α*, of which there are 13 subtypes, IFN-*β*, IFN-*ε*, IFN-*κ*, IFN-*ω*, IFN-*δ*, IFN-*ζ*, and IFN-*τ*.^1^ All IFN-I subtypes act via the broadly expressed IFN-I receptor (IFNAR) complex to activate JAK1/TYK2 kinases, activating STAT transcription factors that induce an antiviral transcriptional state.^2^ The ~2000 genes that are positively regulated by IFN signaling are collectively termed interferon-stimulated genes (ISGs) and include numerous pathogen detection and effector proteins.^3^ In recent years, neurodegenerative diseases have been shown to mimic this antiviral transcriptional signature despite the absence of overt viral infection. In Alzheimer’s disease (AD), the severity of the disease at death is correlated with ISG expression and with levels of IRF7, a transcription factor responsible for the expression of IFN-I itself.^4,5^

AD is a dual proteinopathy, characterized by the presence of two aggregated protein species, extracellular amyloid plaques, and cytoplasmic assemblies of tau.^6^ Under the prominent amyloid cascade hypothesis,^7^ aggregation of amyloid beta (A*β*) precedes and causes tau aggregation and downstream neurodegenerative processes. The mechanisms by which A*β* exerts these effects on tau are not known, but the innate immune system is implicated. Tau pathology in the absence of amyloid plaques can cause neurodegeneration, most strongly evidenced by point mutations in MAPT, the gene that encodes tau that leads to dominantly inherited forms of frontotemporal dementia.^8^ Furthermore, tau pathology in AD is negatively correlated with cognitive function at the time of death.^9^ These observations are consistent with a model wherein tau pathology is a central but downstream driver of neurodegeneration in AD.

The manner by which tau pathology spreads within affected brains is a matter of active research. It has been demonstrated that tau assemblies can be taken up from the extracellular space to neurons following interaction with receptor LRP1 and heparan sulfate proteoglycans at the cell surface.^10^ These assemblies enter the cytosol and can cause seeded aggregation of native tau pools.^11–13^ This mechanism could explain the progressive accumulation of tau over time and shares mechanistic similarities with prion and virus replication.

Recent studies have demonstrated that A*β* is an agonist of the IFN-I pathway.^4,5,14,15^ Transgenic mice that accumulate A*β* in their brains display evidence of ISG expression.^5^ Moreover, the treatment of microglia, the major site of pathogen detection in the brain, with A*β* fibrils, can promote the production of IFN-I.^14,16^ Tau fibrils may also promote microglial IFN-I production, partially by promoting the release of mitochondrial deoxyribonucleic acid (DNA) into the cytosol.^17^ However, the effects of IFN-I on the development of tau pathology are unclear. In this study, we used ex vivo and in vivo models to test the effect of IFN-I signaling on tau pathology. Treatment of neural cultures with polyI:C, a molecular pattern that mimics viral nucleic acids, evoked a potent cytokine response that enhanced seeded aggregation of tau. We observed that IFN-I was the principal driver of this response, as neural cultures lacking the ability to respond to IFN-I were protected from the pro-aggregant effects of polyI:C. Pharmacological antagonists were able to reverse these effects, demonstrating the requirement of canonical IFNAR signaling in the response. IFN-I-induced vulnerability was intrinsic to neurons and occurred after entry of tau assemblies to the cell interior, suggesting that the neuronal cytosol, the main site of tau expression and aggregation, is the site of IFN-induced susceptibility. In animal models of tau pathology, ablation of the IFN-I response by knockout of *Ifnar1* substantially reduced pathological tau burden. The results demonstrate that inflammatory cytokines render neurons susceptible to seeded tau aggregation and that IFN-I is responsible for the majority of this effect. Given the established prominence of IFN-I in the AD brain, our results implicate IFN-I as an accelerant to neurodegenerative processes and as a potential link between inflammatory stimuli and tau pathologies.

## 2 Methods

### 2.1 Mouse lines

All animal work was licensed under the UK Animals (Scientific Procedures) Act 1986 and approved by the Medical Research Council (MRC) and Animal Welfare and Ethical Review Body (AWERB). P301S-tau transgenic mice that had been extensively backcrossed to the C57BL/6 background were obtained from Dr. Michel Goedert, wildtype (WT) mice in this study refer to C57BL/6 mice. *Ifnar1*^−/−^ mice (B6.129S2-Ifnar1tm1Agt/Mmjax) were crossed with P301S-tau transgenic mice through extensive backcross. Male and female mice were used in the study and humanely sacrificed by cervical dislocation. Sample sizes were chosen based on previous studies with the same P301S-tau mouse line.^18,19^ No animal exclusions were made.

### 2.2 Primary mixed neural cultures

Primary mixed cortical and hippocampal neural cultures were isolated as described elsewhere.^20,21^ Cortices/hippocampi were isolated from postnatal day 1 to 2 pups from TgP301S mice. Meninges were removed and cortices and hippocampi digested with trypsin (0.25% wt/vol) and DNAse (0.1% wt/vol, Thermo Fisher Scientific, 50-443-716). Neurons were dissociated by trituration and plated onto precoated poly-L-lysine (0.1 mg/mL, R&D Systems, Cultrex 3438-100-01) plastic ware at a density of 30,000/well in 96 wells in plating medium consisting of Neurobasal Plus (Gibco, A3582901), GlutaMAX (1 mM, Gibco, 35050061), horse serum (10%), penicillin-streptomycin (1%), and 1X B-27 Plus growth factor supplement (Gibco, A3582801). Three hours after plating, medium was exchanged for maintenance medium (plating medium devoid of horse serum). At DIV6, the cultures were topped up with maintenance medium (50% volume). When L-leucine methyl ester (LME)-based microglia depletion was conducted, at DIV6, 15 mM LME was added to the medium for 4 h, and a full medium change into maintenance medium was conducted subsequently (as described in ref. [22]). When PLX3397-based microglia depletion was conducted, 2 *μ*M PLX3397 (SelleckChem, S7818) was added to the medium at DIV0 and supplemented repeatedly every 48/72 h until DIV14.

### 2.3 Cortical/hippocampal neuronal cultures

Primary cortical/hippocampal neuronal cultures were isolated from embryonic day 15.5 pups from TgP301S mice. Meninges were removed, as were cerebellum and olfactory bulbs. Cortices were placed in an enzyme solution consisting of Neurobasal Plus containing papain (1.24 mg/mL, Sigma-Aldrich, P3125) and L-cysteine (0.32 mg/mL, Sigma-Aldrich, C7352) for 20 min at 37°C, before being placed in inhibitor solution containing trypsin inhibitor (1 mg/mL, Sigma-Aldrich, T9128) and BSA (1 mg/mL, Sigma-Aldrich A9647) for 1 min, followed by the same solution at 10X concentration. Cortices were then placed in a dissociation solution containing DNAse (5 *μ*g/mL) and titrated. The suspension was left to settle for 2 min and the supernatant spun for 4 min at 400 rpm. The resulting supernatant was aspirated and the pellet resuspended in maintenance medium (as above). The spin was repeated and the final pellet was resuspended in maintenance medium. After counting, cells were plated at 30,000/well in poly-L-lysine-coated plates in maintenance medium (above). After 30 min, a full medium change into maintenance medium was conducted to remove dead cells. AraC (Sigma, C6645) was added to cultures at DIV0, 5, and 10 at 1 *μ*M.

### 2.4 Production of recombinant tau assemblies

The expression, purification, and aggregation of recombinant human 0N4R tau bearing the P301S mutation was performed as described previously^23–25^ with some modifications. pRK172 plasmid carrying the coding DNA sequence for human P301S 0N4R tau was modified to insert a 6xHis-tag at the C terminus with a TEV cleavage site. The plasmid was transformed into BL21(DE3) pLysS *E. coli* cells, plated onto Luria broth plates containing ampicillin and incubated at 37°C overnight. Colonies were resuspended in 2X Tryptone Yeast broth containing carbenicillin (100 *μ*g/mL), and cultures were grown in a shaking incubator at 37°C until OD0.6 was reached, at which point 0.5 mM isopropyl *β*-D-thiogalactoside was added. Cultures were incubated overnight, shaking at 16°C. Bacterial pellets were collected through centrifugation at 5000 × *g* for 10 min and resuspended in lysis buffer containing 25 mM HEPES pH 7.4, 300 mM NaCl, 20 mM Imidazole, 1 mM phenylmethanesulfonyl fluoride, 1 mM Benzamidin, 14 mM *β*-mercapto-ethanol, 1 mM NP-40, and one protease inhibitor tablet (per 50 mL). After boiling 10 min and being placed on ice for 10 min, ultracentrifugation was carried out at 100,000 × *g* for 50 min. The supernatant was passed through a nickel-affinity column and eluted with elution buffer containing 25 mM Hepes pH 7.4, 300 mM NaCl, and 300 mM imidazole (without protease inhibitors), then fractions were concentrated using a 10 kDa cut-off filter (Amicon Ultra, Millipore) and dialysed three times to remove imidazole, discarding the flow-through. The solution was incubated overnight at 4°C with His-TEV protease (ratio of 10,000 unit TEV protease:tau, 100 mg:1 mg) on a roller and loaded into a second nickel-affinity column. The flow-through was concentrated with a 10 kDa cut-off filter until 1 mL protein solution was obtained, which was run on a HiLoad 16/600 Superdex 200 pg. Fractions were pooled and concentrated to >3 mg/mL using AmiconUltra tubes with a 10 kDa cut-off, (3000 × *g*, 30 min ×5), then diluted in 1X Phosphate-Buffered Saline (PBS) making aliquots of monomeric tau, which were snap frozen and stored at −80°C. At each stage, 15 *μ*L supernatant was removed and used for SDS-PAGE analysis.

Assemblies were prepared by the addition of heparin, as described in ^20^ using tau at 60 *μ*M in the presence of 20 *μ*M heparin, protease inhibitor, 2 mM dithiothreitol (DTT), and 1X PBS, shaking at 37°C for 3 days. Assemblies were sonicated on ice for 15 s and diluted in 1X PBS making aliquots of 20 *μ*M tau assemblies, snap frozen, and stored at −80°C. To confirm aggregation, 50 *μ*L reactions were made up using 7.5 *μ*M tau, 2.5 *μ*M heparin,2 mM DTT, 10 *μ*M ThioflavinT, and protease inhibitor. Each reaction was pipetted into a well of a black 96-well plate that was coated with film to prevent evaporation. The plate was shaken at 37°C for 3 days in an illuminometer (Clariostar), and the fluorescence spectra of ThioflavinT were recorded (excitation and emission wavelength 440 and 510 nm, respectively).

### 2.5 Primary mixed neural culture seeding assay

For seeding assays, treatments, for example, polyI:C (Sigma, P9582), IFN*α* (Sigma, IF009), and IFN*β* (Sigma, IF011), were applied to cultures in the medium at DIV6, 8, 10, and 12, and tau assemblies were supplemented in maintenance medium at DIV7 at 50 nM unless otherwise stated. Repeated treatment with alternative IFN-I agonist LPS from *E. coli* (Sigma, L2630, 1 *μ*g/mL and 100 ng/mL) was found to be toxic and not used in the seeding assays. Cultures were incubated until DIV14 at (37°C, 5% CO_2_) and fixed with 100% methanol, nuclei stained with Hoechst (Life Technologies 33342), and immunofluorescently labeled with MAP2 (Abcam, ab5392), Iba1 (Abcam, ab178846), glial fibrillary acidic protein (GFAP) (Sigma-Aldrich, G3893), AT8 (Thermo Fisher Scientific MN1020), or pS422 (Abcam, ab79415) antibodies. Additional stains included STAT1 (CST, 9172), and IFITM3 (Proteintech, 11714-1-AP). To block IFN-I signaling, cells were pre-incubated with *α*IFNAR1 blocking antibody (InVivoMAb, BioXcell, BE0241) or control IgG (9C12, made in-house) for 1 h before treatment with IFN*β*. Stained neurons were subject to high-content microscopy imaging (10X, Nikon Ti2), and tau aggregation was quantified by AT8-positive or pS422-positive puncta per field normalized to DAPI count/field. A binary mask was created to threshold for Iba1+ and GFAP+ cells, and the number of Iba1+ cells/area covered by GFAP+ cells was quantified using NiS Elements software. For all analyses, five sample replicates were created. Five images/well of a defined region of interest were analyzed for seeding and GFAP+ and Iba1+ cells. Immunoblotting was conducted on samples harvested at DIV7 after an overnight incubation with stimulus. For cytokine analysis, supernatants were collected from cultures at DIV14 prior to fixing unless otherwise stated.

### 2.6 Neuronal tau entry assay

Neuronal entry assays were performed as previously described.^20^ Briefly, neuronal cultures were infected at DIV2 with AAV1/2 hSyn::-eGFP-P2A-LgBiT-nls particles at a multiplicity of 50,000 genome copies per cell. Treatment with polyI:C (2.5 *μ*g/mL), IFN-*α* (50 U/mL), and IFN-*β* (50 U/mL) was performed overnight, while treatment with 2 mM methyl-*β*-cyclodextrin (M*β*CD; Sigma-Aldrich, C4555) was performed for 2 h on DIV7. 50 nM tau-HiBiT assemblies were added to the medium. Cytosolic entry was quantified after 1 h, followed by incubation for 42 min with PrestoBlue Cell Viability Reagent according to the manufacturer’s instructions (Thermo Fisher Scientific). Fluorescence intensity was quantified (excitation 540 to 570 nm; emission 580 to 610 nm) using the ClarioSTAR microplate reader (BMG Labtech). Total viable cells per well were calculated using a standard curve of viable cells per well and adjusted for background fluorescence. The luminescent signal was then normalized to the total viable cells per well and expressed as fold change.

### 2.7 Supernatant cytokine analysis

Individual cytokines (IFN-*β*, CCL3) were quantified in supernatants from mixed or microglia-depleted neuron cultures using DuoSet Sandwich ELISA kits. Dilutions were individually optimized for each cytokine. To analyze supernatants after polyI:C treatment, after overnight incubation, supernatants were run undiluted on Luminex Procarta Plex Mouse Immune Monitoring Panel (48-plex) using media from untreated cells as a control.

### 2.8 Immunofluorescence

For mixed neural cultures, cells were blocked in a 2% BSA (1 h, 22°C) before primary antibodies were diluted in the same buffer and incubated with cells overnight at 4°C. Cells were washed three times in 1X PBS before Alexa Fluor-conjugated secondary antibodies were added to the blocking buffer (1 h, 22°C). Cells were washed three times in 1X PBS and incubated with Hoechst (Life Technologies 33342) in 1X PBS (15 min, 22°C) before imaging.

### 2.9 Western blotting

Cells were lysed in a buffer of 1x Tris-Buffered Saline (TBS), 1% Triton X-100, 1X Protease inhibitors (Halt), and 1X Phosphatase inhibitors (Halt). Lysates were clarified by microcentrifuge (15,000 × *g*, 15 min, 4°C) and transferred to new tubes, to which appropriate volumes of 4X NuPAGE LDS sample buffer containing 2 mM *β*-mercaptoethanol (Thermo Fisher Scientific) were added. Samples were heated (95°C, 5 min) and resolved using NuPAGE Bis–Tris Novex 4% to 12% gels (Life Technologies). Proteins were transferred to a 0.2-*μ*m PVDF membrane using the Transblot Turbo Transfer System (Bio-Rad). Membranes were blocked with 3% goat serum and 0.5% fish gelatin in TBS–0.1% Tween-20 before incubation with primary antibodies (4°C, o/n), CypB: sc-130626, SCB). Membranes were probed with appropriate secondary HRP- or DyLight/Alexa Fluor-conjugated antibodies (1 h, 22°C). Membranes were washed in TBS–0.1% Tween-20 after primary/secondary antibody incubation and imaged (ChemiDoc, Bio-Rad). Capillary gel electrophoresis (CGE) western blot was conducted using Jess, Protein Simple, BioTechne (primary antibodies: Tau12; MAB2241-KC; Millipore, GAPDH: 2275-PC-10; Bio-Techne).

### 2.10 Organotypic hippocampal slice cultures (OHSC)

OHSCs were prepared, seeded, stained, and immunoblotted as previously described.^23^ Briefly, brains from day 7 *Ifnar1*^+/+^ P301S-tau or *Ifnar1*^−/−^ P301S-tau transgenic pups were extracted in a slicing medium (EBSS + 25 mM HEPES) on ice. After bisection upon the midline using a scalpel, the medial surface was attached to the stage of a Leica VT1200S Vibratome using cyanoacrylate (Loctite), and sagittal slices of 300 *μ*m were prepared. Hippocampal slices were dissected using sterile needles and cultured on a 0.4-*μ*m pore size (Millipore) membrane in six-well plates in 1 mL culture medium (50% MEM with GlutaMAX (Gibco,41090036), 18% EBSS (Gibco, 24010043), 6% EBSS + D-glucose, 1% penicillin-streptomycin (Gibco, 15140122), 0.06% nystatin (Gibco, 15340029), and 25% horse serum (Gibco, 26050070). Slices were maintained at 37°C, 5% CO_2_ in a humid atmosphere. IFN-*α* or polyI:C was applied to the underside of the slices in 1 mL culture medium on day 13 before the addition of 100 nM tau assemblies on top of the slices on day 14. IFN-*α* or polyI:C was reapplied every 3 days in a half medium change. Three weeks after challenge, OHSCs were fixed in 4% paraformaldehyde (PFA), permeabilized in 0.5% Tween-20, and blocked and immunofluorescently labeled in a buffer of 3% goat serum in 1X PBS.

### 2.11 Tau pathology and glial analysis in aged mice

Murine brain hemispheres were immersion-fixed for 48 h in 4% PFA with 1X PBS, then dehydrated and cryoprotected in 30% sucrose in 1X PBS for an additional 3 days. The hemisphere was then rapidly frozen in 5-methyl butane, and 25-*μ*m-thick sagittal sections were serially cut through the entire hemisphere. Three sections per mouse brain (at least 300 *μ*m apart) were stained for semi-quantitative immunohistochemistry. Briefly, brain sections were permeabilized using 0.3% Triton X-100 in 1X PBS and blocked in 3% horse serum in the same solution (1 h, 22°C). Sections were incubated overnight with AT8-Biotin (Thermo Fisher Scientific, MN1020B) and NeuN (Abcam, ab177487), GFAP (Sigma, G3893), or Iba1 (Abcam) in 2% horse serum in permeabilization buffer. Sections were washed in permeabilization buffer and incubated with Streptavidin-conjugated Alexa Fluor 647 (2 h, 22°C) and Hoechst 33342 in 1X PBS (10 ug/mL, Thermo Fisher Scientific, 15 min). After mounting, sections were evaluated using a Nikon Ti2 microscope and pathology/microgliosis quantified blinded. Quantification of AT8-immunopositive cell bodies in the cerebral cortex was calculated manually, and the area of AT8+ staining in the brainstem was quantified using a threshold-based binary mask in ImageJ. Sections were rehydrated in 1X PBS before mounting. To stain sections for insoluble tau pathology, a pentameric form of formyl thiophene acetic acid (pFTAA) was used as described in Brelstaff et al.^26^ and incubated with sections in 1X PBS (1:500) for 30 min, before washing with 0.1% Sudan Black (Sigma, 199664) in 50% EtOH for 10 min. Quantification of Iba1+ microglia was calculated manually – three overlapping 638 *μ*m^2^ FOV were randomly selected across the cerebral cortex (sagittal plane), and the number of Iba1+ microglia was quantified per FOV. To quantify the GFAP+ fraction in the hippocampus, three sagittal sections per mouse (> 200 *μ*m apart) were stained. Sections were selected based on the morphology of the hippocampus to match the sagittal structure depicted in the Allen mouse brain atlas at positions 105 to 155 (https://mouse.brain-map.org/static/atlas). Z-stacks of the entire hippocampus were collected. Three or four FOVs (452 *μ*m^2^) were randomly selected across the CA1 and CA2 regions of the hippocampus for analysis. A binary mask was created for each stain on ImageJ, and thresholding analysis was used to measure the percent area fraction.

### 2.12 Cytokine analysis of aged mice

Murine brain cortexes were homogenized in a lysis buffer of 50 mM Tris HCl pH7.4, 150 mM NaCl, 1X protease, and phosphatase inhibitors (Halt) and then incubated with Triton X-100 at 1% for 20 min. After spinning down cellular debris (15,000 × *g*, 10 min), the protein concentration of the lysate was quantified by BCA assay (Biovision) and diluted to 10 mg/mL for analysis with Luminex Procarta Plex Mouse Immune Monitoring Panel (48-plex). Cytokines that were universally not detected (either due to technical limitations or biological level) included TNF-*α*, IFN-*α*, IFN-*γ*, and IL-6 and are not shown.

### 2.13 Seeding assay in HEK293

HEK293 tau-venus stably expressing human P301S 0N4R tau-venus was described previously.^27^ These cells were maintained at 37°C at 5% CO_2_ in complete DMEM (C-DMEM) with 10% (vol/vol) FCS, 100 U/mL penicillin, and 100 *μ*g/mL streptomycin. The seeding assay using spinal cord homogenate from P301S or *Ifnar1*^−/−^ P301S-tau mice was carried out as described previously.^27^ Spinal cords were homogenized using a benchtop homogenizer in 1X PBS. Briefly, HEK293 P301S tauvenus cells were plated at 25,000 cells per well in black 96-well plates precoated with poly D-lysine in 50 *μ*L OptiMEM (Thermo Fisher Scientific). Spinal cord homogenate was diluted in 50 *μ*L OptiMEM (Thermo Fisher Scientific) and added to cells with 0.5 *μ*L per well Lipofectamine 3000. After 1 h, 100 *μ*L c-DMEM was added to each well to stop the transfection process. Cells were incubated at 37°C for 48 h and subsequently fixed in 4% PFA. After staining with Hoechst, tau aggregation in the cells was quantified using high-content image acquisition and analysis (Nikon Ti2, NiS Elements). The percentage of seeded cells was calculated by the number of tau-venus puncta divided by the number of cells.

### 2.14 Image analysis and statistics

Images were acquired on a Ti2-E High Content Microscope (Nikon) or the IncuCyte S3 Live-Cell System. Puncta corresponding to seeded aggregation were quantified using NISElements software. Total puncta per field were normalized to cell count per field via DAPI staining. AT8 staining in OHSCs was segmented into a binary threshold and fields of 150 mm × 150 mm were analyzed for percent AT8 positive area (as described in Miller et al.^23^).

The data in all graphs are represented as the mean ± SD. Data were analyzed via the Kruskal–Wallis test by ranks, unless they was determined to be normally distributed, in which case a one-way ANOVA was employed. Differences between the two means were tested by the unpaired Student *t* test with Welch’s correction. All statistics were carried out in GraphPad Prism Version 9.

## 3 Results

### 3.1 Innate immune stimulation drives seeded tau aggregation via IFN-I

**FIGURE 1:**
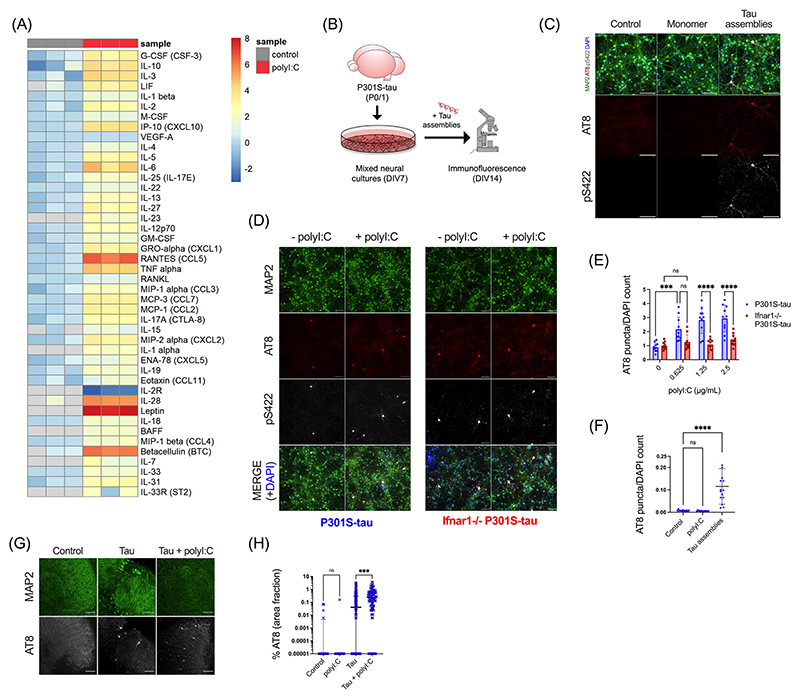
PolyI:C drives seeded tau aggregation in a type-I IFN-dependent manner. Mixed neural cultures were prepared from P301S-tau transgenic mice. (A) Cytokines were measured in the supernatant of cultures treated repetitively with polyI:C (2.5 *μ*g/mL) and compared to control untreated cultures; *n* = 3. Data presented are the log2 of cytokine concentration (pg/mL), which was normalized to the average of control concentrations. (B) Schematic of tau seeding assay in primary mixed neural cultures from P0/P1 mouse brain overexpressing human P301S-tau. Recombinant tau assemblies were added at DIV7, and tau aggregation was monitored by immunofluorescence microscopy using antibody AT8 or pS422 at DIV14. (**C**) Tau monomer and assemblies (50 nM) were added to cultures and levels of AT8 and pS422 reactivity measured by immunofluorescence. (D, E) Seeded aggregation in *Ifnar1*^+/+^ P301S-tau and *Ifnar1*^−/−^ P301S-tau cultures treated with/without polyI:C (2.5 *μ*g/mL) and tau assemblies (50 nM); *n* = 5, *N* = 2 independent experiments. (F) Levels of tau aggregation in *Ifnar1*^+/+^ P301S-tau cultures treated with polyI:C (2.5 *μ*g/mL) alone or tau assemblies (50 nM) alone; *n* = 5, *N* = 3 independent experiments. (G) Representative images of seeded tau aggregation in OHSCs from P301S-tau mice treated with tau assemblies (‘Tau’) (300 nM) ± polyI:C (10 *μ*g/mL) and stained with AT8. (H) Quantification of seeded tau aggregation measured by immunofluorescence; slices from *N* = 6 mice. *n* = wells/condition, each containing 30,000 plated cells. All error bars indicate mean ± SD, except for H, where median and interquartile range are presented. Scale bars = 100 *μ*m. Significance calculated by two-way ANOVA for E and Kruskal–Wallis test with Dunn’s correction for F and H. ***p < 0.001; ****p < 0.0001; ns, not significant.

We first sought to establish whether an inflammatory cytokine response would affect the propensity of tau to undergo seeded aggregation. We treated mixed neural cultures from P301S-tau transgenic mice with polyI:C, a mimetic of viral ribonucleic acid (RNA) and a TLR3 agonist. PolyI:C treatment resulted in abundant production of cytokines from neural cultures and included TNF*α*, IL-6, and IL-1*β* (Figure 1A). IFN-*β* was also secreted and IFITM3, a known interferon stimulated gene (ISG), was upregulated, indicating that polyI:C treatment induced an IFN-I response in neural cultures (Figure S1a, S1b). We tested the contribution of this innate immune response to seeded tau aggregation in response to exogenously applied recombinant heparin-assembled tau (Figure 1B). The addition of tau assemblies, but not monomer, resulted in dose-dependent accumulation of intracellular tau species that were stained by the antibody AT8, specific for tau phosphorylated at S202/T205, and anti-pS422, epitopes that are present in hyperphosphorylated tau inclusions in AD brains (Figure 1C, Figure S1c). We observed that treatment of cultures with polyI:C prior to and during challenge with tau assemblies resulted in significantly higher levels of seeded aggregation as measured by staining for hyperphosphorylated tau (Figure 1D,E). However, polyI:C in the absence of exogenous tau assemblies did not induce tau aggregation (Figure 1F). Thus, innate immune stimulation leads to increased vulnerability of neural cultures to seeded tau aggregation.

We next investigated the contribution of IFN-I in mediating the effect of polyI:C on seeded tau aggregation. To test this, we repeated seeding experiments in neural cultures derived from *Ifnar1*^−/−^ mice. Cells of this knockout strain lack the IFNAR and are universally unable to respond to IFN-I. IFN-Is were produced by *Ifnar1*^−/−^ cultures in response to polyI:C irrespective of IFNAR1 expression (Figure S1a), and seeded tau aggregation was induced with a similar dose dependence in both *Ifnar1*^−/−^ and *Ifnar1*^+/+^ cultures (Figure S1d). However, the effect of polyI:C on seeded tau aggregation was abrogated in *Ifnar1*^−/−^ cultures (Figure 1D,E). Whereas polyI:C treatment resulted in a threefold increase in seeded aggregation in *Ifnar1*^+/+^ cultures, no significant change was observed in the *Ifnar1*^−/−^ background. These results demonstrate that IFN-I increases the vulnerability of neurons to seeded aggregation following innate immune stimulation.

We next tested whether intact neural tissue slices exhibited a similar response to polyI:C. We prepared OHSCs from P301S tau transgenic mice, which retain authentic tissue architecture and cell type identity.^23^ Following a period of microglial rounding and cytokine production in response to slicing, we observed a return to baseline after day 10 (Figure S2a-d). Treatment of quiescent OHSCs with tau assemblies after this point in the presence of polyI:C resulted in a substantial increase in seeded aggregation compared to treatment with tau assemblies alone (Figure 1G,H). Thus polyI:C induces a state of enhanced susceptibility to seeded aggregation in physiologically faithful three-dimensional (3D) brain cultures.

### 3.2 Exogenous IFN-I treatment potentiates seeded tau aggregation

**FIGURE 2:**
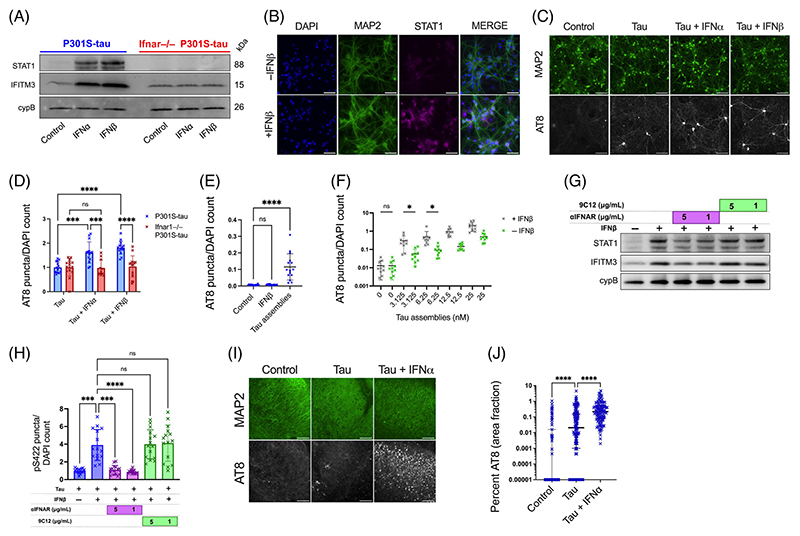
Type-I interferons increase seeded tau aggregation. (A) Mixed neural cultures from *Ifnar1*^+/+^ P301S-tau or *Ifnar1*^−/−^ P301S-tau transgenic mice were treated overnight with IFN-*α* or IFN-*β* and expression of interferon-stimulated genes (ISGs) IFITM3, and STAT1 was measured by western blot. (B) Representative immunofluorescence images of STAT1 and MAP2 staining in cultures from *Ifnar1*^+/+^ P301S-tau mice following overnight incubation with IFN-*β*. (C) Mixed neural cultures from *Ifnar1*^+/+^ P301S-tau or *Ifnar1*^−/−^ P301S-tau mice were treated with tau assemblies (‘Tau’) (50 nM) with/without repetitive treatment of IFN-*α* or IFN-*β* (50U/mL). Seeded tau aggregation was measured by immunofluorescence staining and quantified in (D); *n* = 5, *N* = 3 independent experiments. (E) Seeded tau aggregation was quantified in *Ifnar1*^+/+^ P301S-tau cultures treated with IFN-*β* (50 U/mL) alone or tau assemblies (50 nM) alone; *n* = 5, *N* = 3 independent experiments. (F) Tau assemblies were titrated onto *Ifnar1*^+/+^ P301S-tau cultures treated with IFN*β* (50 U/mL) and seeded aggregation quantified; *n* = 5, *N* = 2 independent experiments. (G) *Ifnar1*^+/+^ P301S-tau cultures were treated with a type-I IFN receptor blocking antibody (*α*IFNAR) or a non-targeting IgG isotype control antibody, anti-adenovirus 9C12 (1 h at 5 *μ*g/mL or 1 *μ*g/mL), before overnight treatment with IFN-*β* (50 U/mL). Expression of IFITM3 and STAT1 was measured by western blot. (H) Seeded tau aggregation was quantified in *Ifnar1*^+/+^ P301S-tau cultures pretreated with IFN-*β* and *α*IFNAR or 9C12 (1 h at 5 *μ*g/mL or 1 *μ*g/mL) and seeded with tau assemblies; *n* = 5, *N* = 2 independent experiments. (I) Representative images of seeded tau aggregation in OHSCs from P301S-tau mice treated with tau assemblies (1000 nM) ± IFN-*α* (50 U/mL) and stained with AT8. (J) Quantification of seeded tau aggregation measured by immunofluorescence; slices from *N* = 6 mice. Western blots representative of *N* = 3 independent experiments. *n* = wells/condition, each containing 30,000 plated cells. All error bars indicate mean ± SD, except for H, where median and interquartile range is presented. Scale bars = 100 *μ*m. Significance calculated by two-way ANOVA for D and Kruskal–Wallis test with Dunn’s correction for E, H, and J and without Dunn’s correction in F. *p < 0.05; ***p < 0.001; ****p < 0.0001; ns, not significant.

To further explore these findings, we treated mixed neural cultures with IFN-Is. A concentration of 50 U/mL (~225 pg/mL) was selected to match concentrations obtained following polyI:C treatment (Figure S1a). We observed that treatment with IFN-*α* or IFN-*β* led to upregulation of the ISGs STAT1 and IFITM3 (Figure 2A,B), Figure S3a,b) and increased seeded tau aggregation, similar to the effect observed for polyI:C (Figure 2C,D). IFN-*β* in the absence of exogenous tau assemblies did not induce tau aggregation (Figure 2E). This pro-aggregant effect of IFN-I was observed over a range of concentrations of tau assemblies and potentiated seeding at low nanomolar concentrations (Figure 2F). Importantly, IFN-I treatment of cultures prepared from *Ifnar1*^−/−^ backgrounds resulted in no upregulation of STAT1/IFITM3 and had no significant effect on the seeded aggregation of tau (Figures 2A, 2D). We verified that the effects of IFN-I were due to a specific interaction between IFN and its receptor by using an IFNAR1-specific antibody that antagonizes IFN-I signaling. Treatment with anti-IFNAR1 at 5 and 1 *μ*g/mL reduced ISG upregulation and reversed the effects of IFN-I on seeded tau aggregation, whereas an isotype control IgG did not (Figure 2G,H). Similarly, treatment of OHSCs with tau assemblies in the presence of IFN-*β* resulted in a substantial increase in seeded aggregation compared to treatment with tau assemblies alone (Figure 2I,J). Thus, IFN-Is also have pro-aggregant effects in physiologically faithful 3D brain cultures.

### 3.3 IFN-I vulnerability is intrinsic to neurons

**FIGURE 3:**
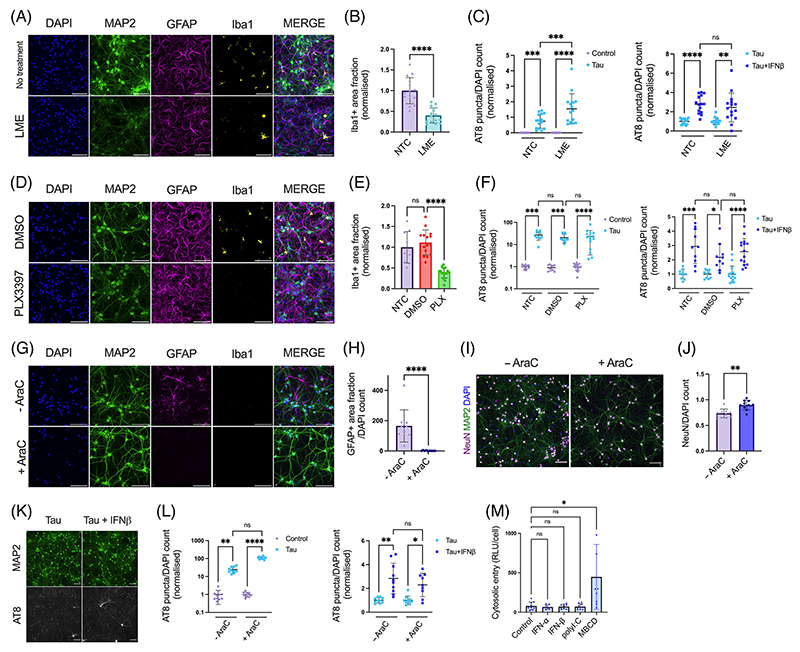
IFN-I vulnerability is intrinsic to neurons. **(A)** Fluorescence microscope images of mixed neural cultures from *Ifnar1*^+/+^ P301S-tau transgenic mice (P0/P1) treated with L-leucine methyl ester (LME, 15 mM for 4 h) revealing reduction of Iba1 positive cell populations. (B) The Iba1+ area fraction was quantified in untreated (NTC) and LME-treated cultures; *n* = 5, *N* = 3 independent experiments. (C) Seeded tau aggregation was quantified in untreated or LME-treated *Ifnar1*^+/+^ P301S-tau cultures, seeded with tau assemblies (50 nM) ± IFN-*β* (50 U/mL); *n* = 5, *N* = 3 independent experiments. (D) Cultures from *Ifnar1*^+/+^ P301S-tau mice treated with DMSO or PLX3397 (PLX, 2 *μ*M) revealing reduction of Iba1-positive cell populations. (E) The Iba1+ area fraction was quantified in untreated and DMSO or PLX3397-treated cultures; *n* = 5, *N* = 3 independent experiments. (F) Seeded tau aggregation was quantified in untreated or PLX3397-treated *Ifnar1*^+/+^ P301S-tau cultures, seeded with tau assemblies (25 nM) ± IFN-*β* (50 U/mL); *n* = 5, *N* = 3 independent experiments. (G) Cortical/hippocampal neuronal cultures were prepared from *Ifnar1*^+/+^ P301S-tau mice at E15.5 using a papain-based protocol. Addition of cytosine arabinoside (AraC, 1 *μ*M) at DIV0/5/7 revealed a complete ablation of GFAP+ cell populations and an absence of Iba1+ cells. (H) The GFAP+ area fraction was quantified in untreated and AraC-treated cultures; *n* = 5, *N* = 3 independent experiments. (I) Representative images of NeuN staining at DIV12. (J) The number of NeuN+ nuclei was quantified by image analysis and values are presented normalized to the DAPI count; *n* = 10 from *n* = 2 independent plates. (K) Representative images and (L) quantification of seeded tau aggregation in AraC treated *Ifnar1*^+/+^ P301S-tau neuronal cultures, seeded with tau assemblies (20 nM) on DIV6 and treated with/without IFN-*β* (25 U/mL), with fixation at DIV12; *n* = 10, *n* = 2 independent plates. (M) Tau entry assay in primary mixed neural cultures from *Ifnar1*^+/+^ P301S-tau mice. Cultures were infected with hSyn::-eGFP-P2A-LgBiT-NLS and treated overnight at DIV6 with polyI:C (2.5 *μ*g/mL), IFN-*α*, IFN-*β* (both 50 U/mL), or methyl-*β*-cyclodextrin (MBCD), (2 mM) for 2 h on DIV7. Cytosolic entry of tau was quantified by luminescence intensity 1 h after addition of tau-HiBiT assemblies to the medium. Cytosolic entry was normalized to the number of viable cells per well; *n* = 3, *N* = 3 independent experiments. *n* = wells/condition, each containing 30,000 plated cells. All error bars indicate mean ± SD. Scale bars = 100 *μ*m. Significance calculated by Mann–Whitney test for B, H and J and Kruskal–Wallis test with Dunn’s correction for C, E, F, L and M. *p < 0.05; **p < 0.01; ***p < 0.001; ****p < 0.0001; ns, not significant.

We next asked which cell types were responsible for mediating the effect of IFN-I on seeded tau aggregation. One possibility is that IFN-I acts directly on neurons to induce a state of enhanced susceptibility to seeding. An alternative explanation is that IFN-I acts on other cell types, for example, microglia or astrocytes, major sources of cytokines in the brain, which in turn enhance susceptibility to seeding in neurons. To test these alternative hypotheses, we used three methods of chemical ablation to variously remove microglia and astrocytes. LME^22^ is a lysomotropic agent that specifically ablated microglia with no discernible effect on astrocytes or neurons (Figure 3A,B), Figure S4a-e). Similarly, PLX3397, a CSF-1R antagonist which has been used to successfully deplete microglia in vivo,^28,29^ reduced microglia with minimal perturbations to the other cell types (Figure 3D,E), Figure S4f-k). Following depletion of microglia using these compounds, we observed that MIP-1*α*, a cytokine of macrophage/microglia origin, was substantially reduced (Figure S4c,h). Treatment of these microglia-depleted cultures with tau assemblies in the presence or absence of IFN-*β* demonstrated that the sensitivity of tau seeding to IFN-I remained intact (Figure 3C, Figure 1F, Figure S4d,j).

We next used cytosine arabinoside (AraC), a nucleotide analog that inhibits cell proliferation by DNA chain termination, to deplete glial cells from embryonic neural cultures. We observed complete removal of GFAP+ cells following AraC treatment (Figure 3G,H). Additionally, no Iba1+ cells were observed. Thus AraC treatment results in cultures that are highly enriched in neurons (Figure 3I,J). Under these conditions, we observed that IFN-I was still able to exert a pro-aggregant effect on seeded tau aggregation (Figure 3K,I Figure S4l,m). Thus, IFN-I can act directly on neurons to enhance sensitivity to seeded tau aggregation.

It was recently demonstrated that the entry of tau assemblies to the cytosol could be rate-limiting to seeding.^20^ We therefore tested whether IFN-I treatment acted to enhance uptake and entry of tau assemblies to the neuronal cytosol using a split-luciferase tau entry assay. We observed that treatment with IFN-I or polyI:C had no effect on cytosolic tau entry, while a positive control treatment, cholesterol extraction using methyl-beta-cyclodextrin (MBCD), increased tau entry (Figure 3M). Taken together, these results suggest that IFN-I increases seeded aggregation by acting on a process in neurons after the entry of tau assemblies to the cytosol.

### 3.4 Genetic depletion of *Ifnar1* reduces tau pathology in vivo

**FIGURE 4:**
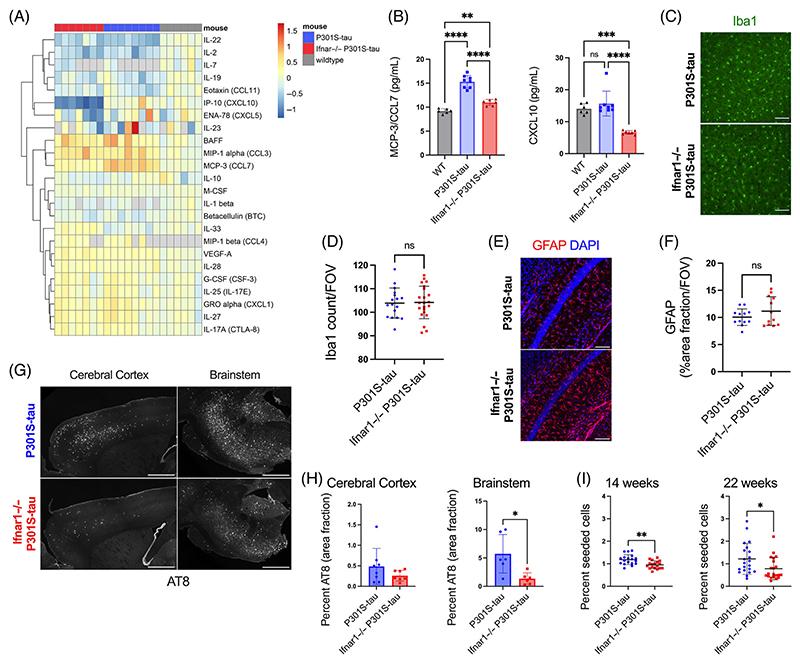
Genetic depletion of Ifnar1 reduces tau pathology in vivo. (A) Cortical cytokine profile of wildtype (*n* = 6 M), *Ifnar1*^+/+^ P301S-tau (*n* = 3 M, *n* = 4 F), *Ifnar1*^−/−^ P301S-tau (*n* = 4 M, *n* = 3 F) mice at 22 weeks of age, measured by Luminex 48-plex assay. Data presented are log2 of cytokine concentration (pg/mL), which was normalized to average of WT controls for each cytokine. Gray = not detected. (B) Replot of selected cytokines CXCL10 and CCL7 observed to be significantly different between *Ifnar1*^+/+^ and *Ifnar1*^−/−^ P301S-tau animals. Subanalysis of male-only mice showed the same effects. (C) Representative images and (D) quantification of Iba1 staining in cerebral cortex of *Ifnar1*^+/+^ P301S-tau and *Ifnar1*^−/−^ P301S-tau mice. Points represent *n* = 3/4 sections/mouse from *Ifnar1*^+/+^ P301S-tau (*n* = 3 M, *n* = 3 F), *Ifnar1*^−/−^ P301S-tau (*n* = 3 M, *n* = 3 F) mice. (E) Representative images and (F) quantification of GFAP staining in hippocampus of *Ifnar1*^+/+^ P301S-tau and *Ifnar1*^−/−^ P301S-tau mice. Points represent *n* = 3 or 4 sections/mouse from *Ifnar1*^+/+^ P301S-tau (*n* = 2 M, *n* = 2 F), *Ifnar1*^−/−^ P301S-tau (*n* = 2 M, *n* = 2 F) mice. (G) Representative images and (H) quantification of AT8 staining in 22-week-old *Ifnar1*^+/+^ P301S-tau and *Ifnar1*^−/−^ P301S-tau brain sections. Points represent average of *n* = 3 sections/mouse (at least *n* = 6 mice per group, *n* = 3 M and *n* = 3 F in each group). (I) Quantification of seeded tau aggregation in HEK293 cells expressing P301S tau-venus, treated with spinal cord homogenate from *Ifnar1*^+/+^ P301S-tau, *Ifnar1*^−/−^ P301S-tau at 14 weeks (*n* = 6 mice per group, *n* = 3 M and *n* = 3 F in each group) and 22 weeks (*n* = 7 mice per group). Points represent *n* = 3 technical replicates per animal. Scale bar = 100 *μ*m for C, E and 1000 *μ*m for G. Significance calculated by one-way ANOVA for B, Welch’s *t*-test for D, F, and I, and Mann–Whitney test for H. *p < 0.05; **p < 0.01; ***p < 0.001; ****p < 0.0001; ns, not significant. Mean and SD are presented.

Having demonstrated that IFN-I can promote seeded aggregation of tau in ex vivo models, we sought to test whether it might have a role in vivo. Transgenic mice expressing human tau-bearing frontotemporal lobar degeneration-causing mutation P301S were used as a model of tau pathology. In this mouse model, neurofibrillary tangles are found to accumulate with age, most substantially in the spinal cord and brainstem, but also affecting the cerebral cortex and hippocampus.^18,30^ P301S-tau transgenic mice were extensively backcrossed to *Ifnar1*^−/−^ mice and central nervous system tissue was analyzed at 22 weeks of age, a time point with substantial tau pathology. We quantified the cytokine profile of the cortical lysate of 22-week-old *Ifnar1*^+/+^ P301S-tau, *Ifnar1*^−/−^ P301S-tau, and WT mice using a quantitative multiplexed protein-level assay. We observed an altered cytokine phenotype in the cortex of mice that expressed P301S-tau compared to WT, including significant upregulation of cytokines BAFF, MIP-1*α*/CCL3, MIP-1*β*, GRO-*α*/CXCL1, and MCP-3/CCL7 and downregulation of IL-22 and IL-2 (Figure 4A, Figure S5a). Notably, the cytokine profiles of *Ifnar1*^+/+^ P301S-tau and *Ifnar1*^−/−^ P301S-tau mice were broadly similar (Figure S4a). However, significant changes were observed for two IFN-I-regulated cytokines, CCL7 and CXCL10, which were reduced in *Ifnar1*^−/−^ P301S-tau mice to levels similar to or lower than levels in non-transgenic mice (Figure 4B). The number of Iba1+ microglia did not change between *Ifnar1*^−/−^ and *Ifnar1*^+/+^ mice (Figure 4C,D), and the area covered by GFAP+ astrocytes in the hippocampus was unchanged (Figure 4E,F). These data suggest that tau pathology induces an altered cytokine state but that IFNAR-dependent signaling contributes to a limited subset of these cytokines.

We next quantified tau pathology at 22 weeks of age using immunofluorescence staining for hyperphosphorylated tau at the AT8 epitope. AT8+ tau inclusions were present in the cell bodies and axons of NeuN+ neurons, consistent with previous descriptions of this transgenic tau model (Figure S5b). We observed a significant reduction in the area covered by AT8+ tau pathology in the brainstem in the *Ifnar1*^−/−^ P301S-tau mice compared to *Ifnar1*^+/+^ P301S-tau mice (Figure 4G,H). Levels of tau pathology in the brainstem were reduced by approximately 80% upon *Ifnar1* knockout. Cortical tau inclusions exhibited a significant reduction in between-animal variance (*F*-test) of AT8+ tau pathology, without changes in total tau (Figure S5c,d). To confirm that the observed changes in phospho-tau reflected a reduction in fibrillar tau assemblies, we used the pentameric form of formyl thiophene acetic acid (pFTAA), a dye that specifically stains filamentous tau.^26^ We observed a pattern of staining similar to that of immunostaining with AT8, with *Ifnar1* knockout causing a reduction in the number of pFTAA+ cell bodies (Figure S5e,f), without gross changes in NeuN+ neurons (Figure S5g). We next tested whether the reduction in tau pathology was also reflected in the quantity of seed-competent tau species by applying spine homogenates to reporter HEK293 cells expressing P301S tau-venus^27^ (Figure 4I). We observed a statistically significant but relatively minor effect of *Ifnar1* knockout at 14 weeks of age, which increased to a twofold difference in seed-competent tau species at 22 weeks. Given that early tau pathology is necessarily driven by cell-autonomous aggregation, this observation is consistent with our ex vivo observations where IFN rendered neurons susceptible to seeded tau aggregation but did not induce cell autonomous aggregation. These findings provide in vivo support for a model wherein IFN-I drives propagating tau pathology by sensitizing neurons to seeded aggregation.

Supplementary material is available at *Alzheimer’s and Dementia* online.

## 4 Discussion

IFN-I is a key feature of brains undergoing proteinopathy and neurodegeneration.^5,31,32^ In this study, we found that IFN-I sensitized neurons to seeded tau aggregation. Using ex vivo primary neuron/glial culture models of tau aggregation, we found that the innate immune agonist polyI:C acted via type-I IFN to increase the seeded aggregation of tau. The effect of IFN-I was manifest within the cytosol of neurons, was independent of microglia and astrocytes, and could be prevented by pharmacological antagonism of IFN-I signaling. Mice lacking the ability to respond to IFN-I did not differ substantially in the innate immune response to tau, with the exception of chemokines CXCL10 and CCL7, whose expression is driven by IFN-I. However, tau pathology was substantially lower when IFN-I signaling was prevented, with approximately fourfold lower tau pathology in the brainstem in *Ifnar1*^−/−^ versus *Ifnar1*^+/+^ mice. Given the widespread evidence of IFN-I responses in the degenerating brain,^5,31^ our results implicate IFN-I as a potentially important driver of tau pathology. cGAS is a cytosolic DNA sensor upstream of IFN-I production, and the cGAS-STING signaling pathway has been identified as a driver of neurodegeneration.^17,32^ Notably, however, ablation of cGAS in PS19 (P301S-tau) mice preserves synaptic integrity and neuronal health, but without effects on tau pathology.^17^ This contrasts with our results in which tau pathology was significantly reduced following genetic deletion of IFNAR. Furthermore, polyI:C, which does not stimulate the cGAS-STING pathway, increased seeded tau aggregation in our study. Taken together, our results suggest that sensors aside from cGAS that activate IFN-I are important for the accumulation of tau pathology in the degenerating brain.

IFN-I is produced homeostatically at basal levels and at higher levels in response to pathogen and damage-associated molecular patterns. We observed that P301S-tau transgenic animals had an inflammatory cytokine response relative to WT mice but comparatively minor changes in cytokines in P301S-tau transgenic mice were observed between *Ifnar1*^+/+^ and *Ifnar1*^−/−^ genotypes. Thus, tau pathology alone does not induce a widespread inflammatory response via IFN-I. Rather, it appears that the difference in tau pathology between *Ifnar1*^+/+^and *Ifnar1*^−/−^ genotypes relies on low, potentially homeostatic, levels of IFN-I production and not on the broader proinflammatory cytokines that accompany aggregation. Other upstream IFN-I agonists may drive a more potent IFN-I response and may be expected to further exacerbate tau pathology. It is therefore notable that A*β* and, more recently, tau^17^ have been shown to induce a potent IFN-I response in patient, mouse model, and culture-based settings.^4,5,14,15,33,34^ Furthermore, virus infection is a key stimulus for IFN-I production and a risk factor for common neurodegenerative diseases^35^ and rare diseases such as subacute sclerosing panencephalitis, which feature tau pathology.^36^ Our data therefore implicate IFN-I as a potential link between the upstream drivers of inflammation, including A*β* and subsequent tau pathologies, as envisaged in the amyloid cascade hypothesis.^7^

Our results strongly suggest that the enhancement of seeded tau aggregation by IFN-I is internal to neurons. A number of mechanisms may be responsible for such a phenotype. First, IFN-I treatment leads to the upregulation of ISGs, so a single ISG, or a set of ISGs, may be responsible for the increase in seeded tau aggregation. Notably, IFN-I induces widespread phosphorylation changes in the murine brain, driven by kinase families known to phosphorylate tau.^37^ Further, IFN-I, either through ISGs or JAK/STAT signaling directly, may affect the degradation of tau assemblies entering the cell or following their formation in the seeded tau aggregation process. Festa et al. recently showed that the cytokine receptor CCR5 (which binds IFN-I-associated ligands for MIP-1*a*/CCL3, MIP-1*b* /CCL4, and CCL5) promoted tauopathy in in vivo models, partially through the regulation of autophagy.^38^ A second alternative is that IFN-I may promote neuronal activity/hyperexcitability, which has been associated with the development of tauopathy in vitro, by increasing tau release and uptake, and in vivo, using optogenetic approaches.^39,40^ To further understand the mechanism in vivo, cell-type-specific knockdown approaches for *Ifnar1* and transcriptomic approaches will further illuminate the contributions of different cell types on tau pathology. Understanding of these mechanisms may illuminate novel targets for potential therapeutic targeting of the IFN-I response in tauopathies.

Our finding that IFN-I response signaling promotes tau pathology further supports approaches to target neuroinflammation in neurodegenerative disease.

## Supplementary Material

Figures S1-S5

## Data Availability

Raw data were generated at the UK Dementia Research Institute at the University of Cambridge, Department of Clinical Neurosciences. Derived data supporting the findings of this study are available from the corresponding author on request.

## Highlights

- Type-I IFN (IFN-I) promotes seeded tau aggregation in neural cultures.
- IFNAR inhibition prevents IFN-I driven sensitivity to tau aggregation.
- IFN-I driven vulnerability is intrinsic to neurons.
- Tau pathology is significantly reduced in aged P301S-tau mice lacking IFNAR.

## Research In Context

### 1 Systematic review

The authors reviewed the literature using traditional (eg, PubMed) sources, conference abstracts, and presentations. The type-I IFN response is a common feature of Alzheimer’s diseased brains and other tauopathies. However, its influence in the progression of pathological tau accumulation is not understood.

### 2 Interpretation

Our findings describe the IFN-I response as an important driver of tau aggregation in animal models. The IFN-I response may therefore act as a potential link between inflammatory stimuli (including amyloid beta) and subsequent tau pathology.

### 3 Future directions

We propose scientific questions necessary to expand on the finding described, including further understanding of the following: (a) molecular mechanism of IFN-I-induced potentiation of seeded tau aggregation, (b) conservation of the phenotype in human neuronal models of tau pathology, (c) effects of *Ifnar1*^−/−^ on microglial subtypes and tau pathology in models of AD, and (d) pharmaceutical antagonism of IFN-I in P301S-tau transgenic mice.
