# Supplementary material for: The type-I interferon response potentiates seeded tau aggregation and exacerbates tau pathology
Source: Alzheimers Dement. Author manuscript; Available in PMC 2024 Mar 12. (PMC10916982; doi:10.1002/alz.13493)
Supplement: Figures S1-S5 [file EMS194371-supplement-Figures_S1_S5.pdf]

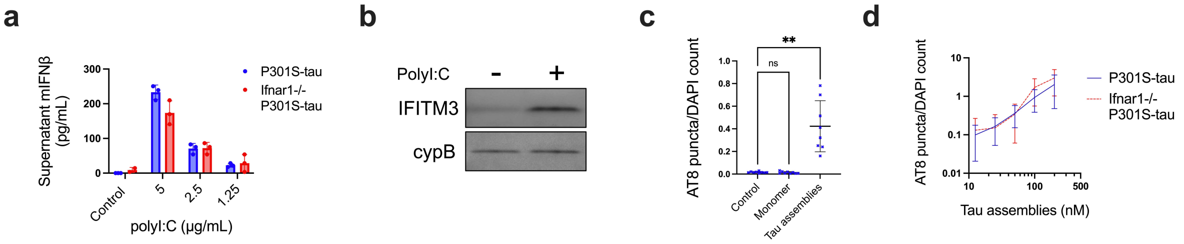

Supplementary Figure 1: **Tau seeding assay in primary mixed neural cultures.** (**A**) IFNβ concentration in the supernatant of *Ifnar1*$^{+/+}$ P301S-tau and *Ifnar1*$^{-/-}$ P301S-tau cultures treated overnight with polyI:C, measured by ELISA; n = 3/genotype. (**B**) Western blot of *Ifnar1*$^{+/+}$ P301S-tau cultures treated overnight with polyI:C (2.5 mg/mL) and probed for ISG IFITM3. Western blot representative of N = 2 independent experiments. (**C**) Tau monomer and assemblies (50 nM) were added to cultures and levels of AT8 reactivity measured by immunofluorescence and quantified by image analysis; n = 4, N = 2 independent experiments. (**D**) Titration of recombinant tau assemblies on cultures from *Ifnar1*$^{+/+}$ P301S-tau and *Ifnar1*$^{-/-}$ P301S-tau mice. Tau assemblies were added at DIV7 and seeded tau aggregation (AT8 epitope) was quantified at DIV14 by image analysis; n = 5, N = 2 independent experiments. ‘n’ = wells/condition, each containing 30,000 plated cells. All error bars indicate mean ± SD. Scale bar = 100 mm. Significance calculated by Kruskal-Wallis test for C. ****P < 0.0001; **P < 0.01; *P < 0.05; ns, not significant.

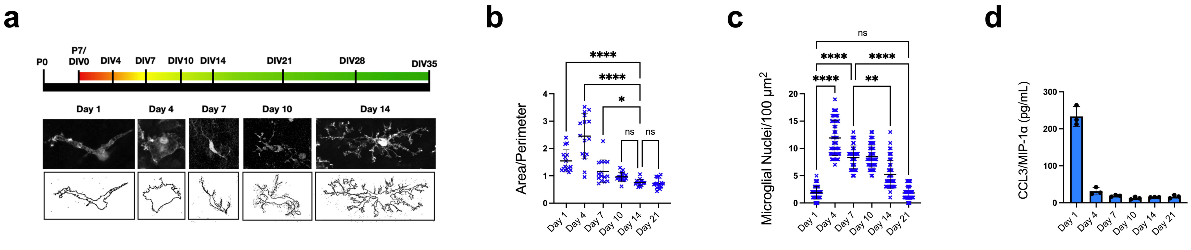

Supplementary Figure 2: **Microglial phenotypes and inflammation in organotypic hippocampal slice cultures.** (**A**) Representative images of Iba1+ microglia staining in organotypic hippocampal slice cultures (OHSCs) from P301S-tau mice, from DIV1 to DIV21. (**B**) Microglial ramification was quantified manually by using a binary mask to calculate perimeter and area and expressed as area/perimeter (n = 15 microglia/timepoint, from N = 3 OHSCs). (**C**) Microglial nuclei number was quantified manually in a FOV of 100 µm^2 (N = 3 OHSCs). (**D**) The concentration of Macrophage Inflammatory Protein 1 alpha (MIP-1α) secreted by OHSCs into the supernatant was determined by ELISA; N = 3 OHSCs/sample; N = 3 independent experiments. Significance calculated by one-way ANOVA for B, C. ****P < 0.0001; **P < 0.01; *P < 0.05; ns, not significant.

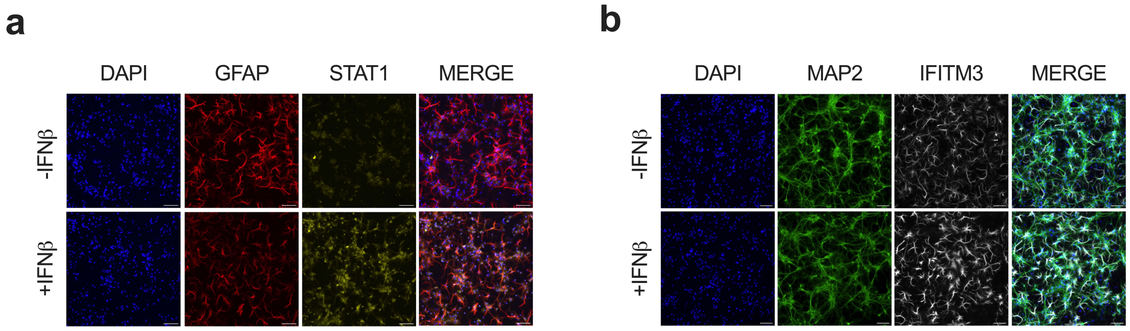

Supplementary Figure 3: **Responses to IFN-I in primary mixed neural cultures.** (**A**) Representative immunofluorescence images in cultures from *Ifnar1*$^{+/+}$ P301S-tau mice following overnight incubation with IFNβ (50 U/mL). Staining for STAT1 and GFAP or (**B**) MAP2 and IFITM3 suggested that both STAT1 and IFITM3 are expressed in astrocytes. Images representative of n = 3 wells/condition, N = 2 independent experiments. Scale bars = 100 μm.

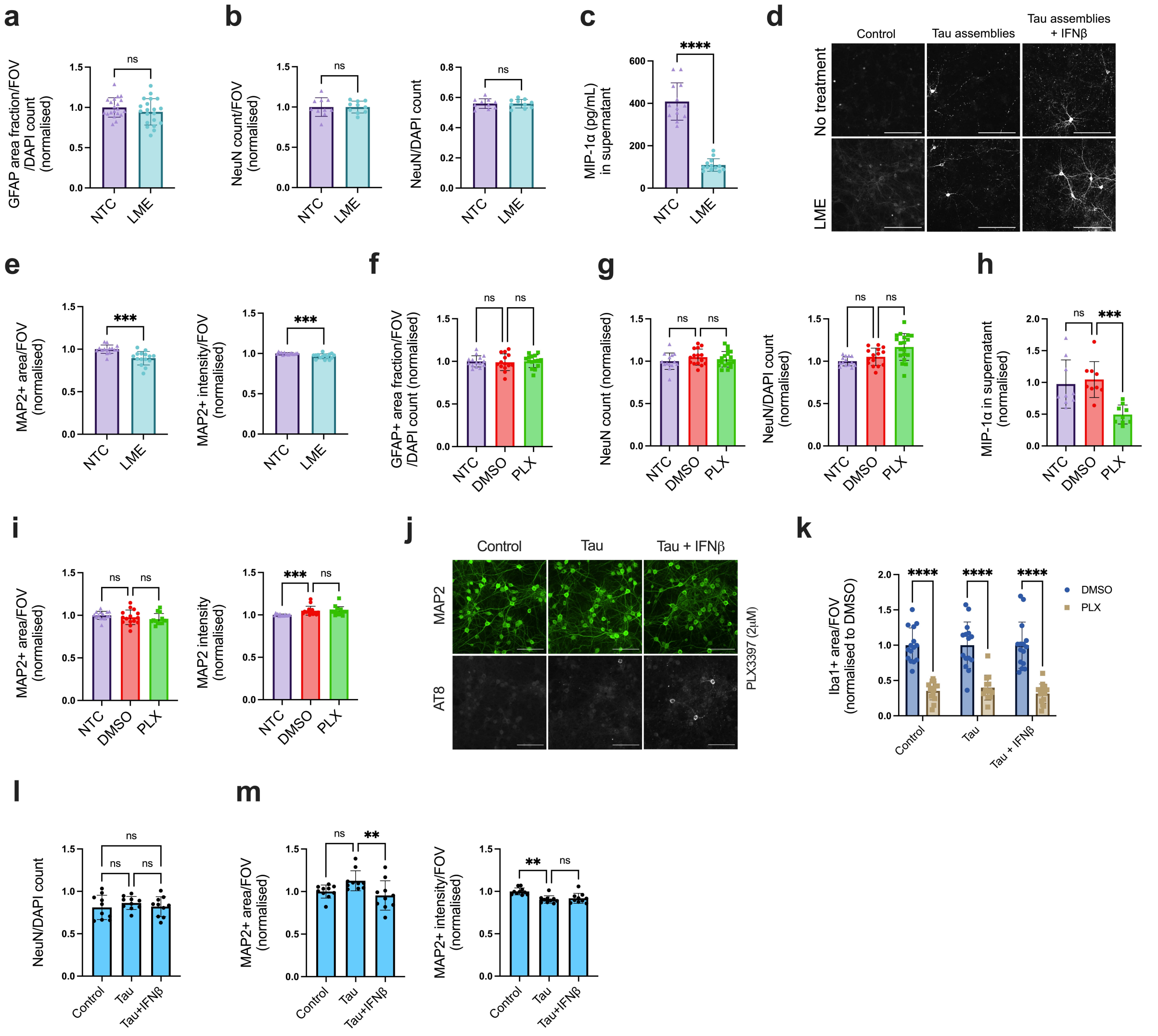

Supplementary Figure 4: **Glial depletion in P301S-tau mixed neural cultures.** (**A**) Quantification of area covered by GFAP+ astrocytes following LME treatment, normalised to DAPI count for each image, and normalised to no-treatment condition (NTC); n = 5, N = 3 independent experiments. (**B**) LME treated cultures were stained for NeuN and the number of NeuN+ nuclei quantified by image analysis. Values are presented normalised to the no-treatment condition and/or the DAPI count; n = 5, N = 2 independent experiments. (**C**) The concentration of Macrophage Inflammatory Protein 1 alpha (MIP-1α) was measured in untreated and LME-treated cultures; n = 3, N = 3 independent experiments. (**D**) Representative image of AT8 staining for seeded aggregation induced by tau assemblies (50 nM) in untreated and LME-treated cultures (15 mM for 4h), in the presence and absence of IFNβ (50 U/mL). (**E**) Quantification of the area fraction covered by MAP2+ cells and MAP2 intensity/FOV in LME-treated cultures, normalised to the no-treatment condition; n = 5, N = 3 independent experiments. (**F**) Quantification of area covered by GFAP+ astrocytes following PLX3397 (PLX, 2µM) treatment, normalised to DAPI count for each image, and normalised to no-treatment condition; n = 5, N = 3 independent experiments. (**G**) The number of NeuN+ nuclei in PLX3397-treated cultures was quantified by image analysis. Values are presented normalised to the no-treatment condition and/or the

DAPI count; n = 5, N = 3 independent experiments. (**H**) The concentration of MIP-1α was measured in untreated and PLX3397-treated cultures; n = 3, N = 3 independent experiments. (**I**) Quantification of the area fraction covered by MAP2+ cells and MAP2 intensity/FOV in PLX3397-treated cultures, normalised to the no-treatment condition; n = 5, N = 3 independent experiments. (**J**) Representative image of MAP2 and AT8 staining for seeded aggregation induced by tau assemblies (25 nM) in untreated and PLX3397-treated cultures, in the presence and absence of IFNβ (50 U/mL). (**K**) Iba1+ area fraction in PLX3397-treated cultures was quantified and normalised to DMSO treated cultures; n = 5, N = 3 independent experiments. (**L**) Quantification of NeuN+ nuclei normalised to the DAPI count and (**M**) area fraction covered by MAP2+ cells and MAP2 intensity/FOV in AraC-treated cultures from E15.5 *Ifnar1*$^{+/+}$ P301S-tau mice, treated with tau assemblies (20 nM) +/- IFNβ (25 U/mL) normalised to the no-treatment condition for M; n = 10 from N = 2 independent plates. 'n' = wells/condition, each containing 30,000 plated cells. All error bars indicate mean ± SD. Scale bars = 100 μm. Significance calculated by Welch's t-test for A, B, C, E, Kruskal-Wallis test for F-I, L, M and two-way ANOVA for K. ****P < 0.0001; **P < 0.01; *P < 0.05; ns, not significant.

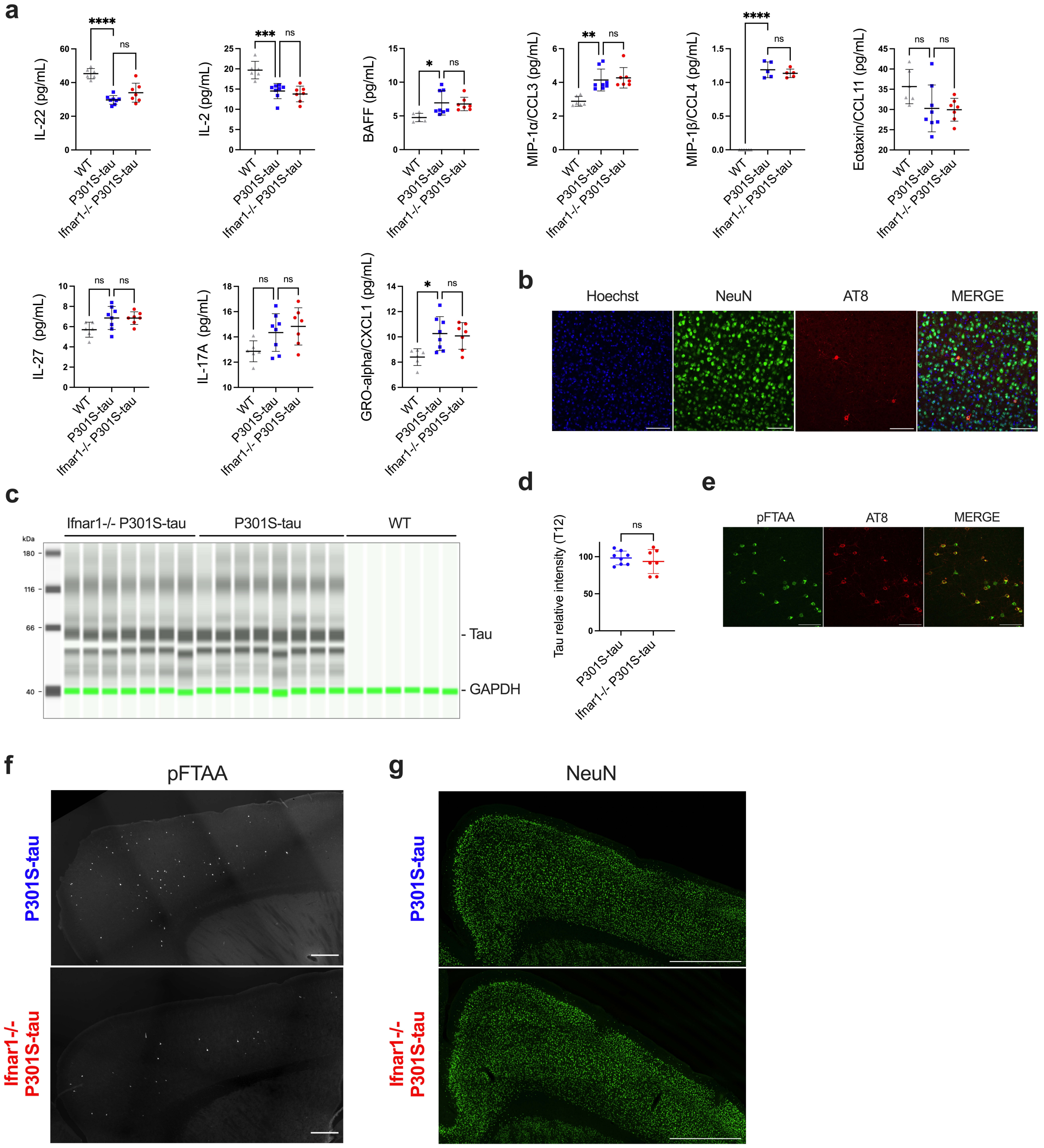

Supplementary Figure 5: Cytokine profile and tau pathology analysis of *Ifnar1*$^{+/+}$ P301S-tau and *Ifnar1*$^{-/-}$ P301S-tau mice. (**A**) Replot of additional cytokines from 48-plex Luminex assay in Fig. 4 comparing cortical cytokine profile of wildtype (n = 6 M), *Ifnar1*$^{+/+}$ P301S-tau (n = 3 M, n = 4 F), *Ifnar1*$^{-/-}$ P301S-tau (n = 4 M, n = 3 F) mice at 22 weeks of age. (**B**) Representative immunofluorescence image from sagittal section of 22 week P301S-tau transgenic mice showing AT8 positive tau inclusions are found in the cell body and axons of NeuN+ neurons in the cerebral cortex. (**C**) Capillary gel electrophoresis western blot of cortical homogenate from *Ifnar1*$^{-/-}$ P301S-tau, *Ifnar*$^{+/+}$ P301S-tau and WT mice using Tau12 (anti-human tau) and GAPDH antibodies, and (**D**) quantification of human tau intensity relative to GAPDH. (**E**) Immunofluorescence images of fibrillar tau staining in sagittal section of 22 week P301S-tau transgenic mice, showing pentameric form of formyl thiophene acetic acid (pFTAA) colocalisation with

AT8 positive tau inclusions in the cerebral cortex. (**F**) Representative images of pFTAA staining in the cerebral cortex of 22 week *Ifnar1*$^{+/+}$ P301S-tau and *Ifnar1*$^{+/+}$ P301S-tau mice. (**G**) Representative image of NeuN staining in the cerebral cortex of 22 week *Ifnar1*$^{+/+}$ P301S-tau and *Ifnar1*$^{+/+}$ P301S-tau mice at 22 weeks. Scale bar = 100 µm for B, C, 200 µm for D and 1000 µm for E. Significance calculated by one-way ANOVA for A. ****P < 0.0001; **P < 0.01; *P < 0.05; ns, not significant.
